# MicroRNA-193a-5p Regulates the Synthesis of Polyunsaturated Fatty Acids by Targeting Fatty Acid Desaturase 1 (*FADS1*) in Bovine Mammary Epithelial Cells

**DOI:** 10.3390/biom11020157

**Published:** 2021-01-25

**Authors:** Yongliang Fan, Abdelaziz Adam Idriss Arbab, Huimin Zhang, Yi Yang, Xubin Lu, Ziyin Han, Zhangping Yang

**Affiliations:** 1 College of Animal Science and Technology, Yangzhou University, Yangzhou 225009, China; dx120170088@yzu.edu.cn (Y.F.); arbabtor@yahoo.com (A.A.I.A.); minmin-911@163.com (H.Z.); dx120180094@yzu.edu.cn (X.L.); ZiyinHan@126.com (Z.H.); 2 Joint International Research Laboratory of Agriculture & Agri-Product Safety, Ministry of Education, Yangzhou University, Yangzhou 225009, China; 3 Jiangsu Co-Innovation Center for the Prevention and Control of Important Animal Infectious Diseases and Zoonoses, College of Veterinary Medicine, Yangzhou University, Yangzhou 225009, China; yangyi@yzu.edu.cn

**Keywords:** bovine milk, polyunsaturated fatty acids, MicroRNA-193a-5p, *FADS1*, bovine mammary epithelial cells

## Abstract

Cardiovascular diseases (CVDs) are seriously threatening to human life and health. Polyunsaturated fatty acids (PUFAs) are known for their role in preventing CVDs. It is beneficial to population health to promote the content of PUFAs in bovine milk. In recent years, limited research based on molecular mechanisms has focused on this field. The biological roles of numerous microRNAs (miRNAs) remain unknown. In this study, a promising and negatively correlated pair of the miRNA (miRNA-193a-5p) and a fatty acid desaturase 1 (*FADS1*) gene are identified and screened to explore whether they are potential factors of PUFAs’ synthesis in bovine milk. The targeted relationship between miRNA-193a-5p and *FADS1* in bovine mammary epithelial cells (BMECs) is demonstrated by dual luciferase reporter assays. qRT-PCR and western blot assays indicate that both the expression of mRNA and the protein *FADS1* show a negative correlation with miRNA-193a-5p expression in BMECs. Also, miR-193a-5p expression is positively correlated with the expression of genes associated with milk fatty acid metabolism, including ELOVL fatty acid elongase 6 (*ELOVL6*) and diacylglycerol O-acyltransferase 2 (*DGAT2*). The expression of fatty acid desaturase 2 (*FADS2*) is negatively correlated with miR-193a-5p expression in BMECs. The contents of triglycerides (TAG), eicosapentaenoic acid (EPA), docosapentaenoic acid (DPA) and docosahexaenoic acid (DHA) have a significant positive correlation with the expression of *FADS1* and a significant negative correlation with the expression of miR-193a-5p in BMECs. For the first time, this study confirms that miRNA-193a-5p regulates PUFAs metabolism in BMECs by targeting *FADS1*, indicating that miRNA-193a-5p and *FADS1* are underlying factors that improve PUFAs content in bovine milk.

## 1. Introduction

Fats are some of the major nutrients in bovine milk and are mainly triglycerides (TG) containing saturated fatty acids (SFAs), unsaturated fatty acids (UFAs) and several minor compounds [1,2]. Although milk fat rich in SFAs has been proposed as a potential risk factor for cardiovascular diseases (CVDs) [3,4], accumulating evidence shows the concern is unnecessary, whether it is for people who consume low-fat milk or high-fat milk [5,6]. A total of 17 fatty acids found in bovine milk, such as trans-palmitoleic acid and conjugated linoleic acid (LA), may prevent excessive obesity [7]. Duda et al. [8] found that dietary supplementation with n-3 Polyunsaturated fatty acids (PUFAs) attenuated pressure overload-induced left ventricle dysfunction in a male rat. Mölenberg et al. [9] showed that shifting the fatty acid composition of the diet toward a higher proportion of UFAs might lower the mortality risk of CVDs in drug-treated patients with cardiac disease. Through detecting the levels of PUFAs and blood lipids of 156 healthy individuals, Wang et al. [10] demonstrated that n-3 fatty acids might adjust blood lipid levels and lower triglyceride levels. In addition, Fonollá et al. [11] demonstrated that daily intake of milk enriched with fish oil rich in n-3 PUFAs, oleic acid, and vitamins improved the nutritional status and cardiovascular risk markers of volunteers, whereas skimmed milk and semi-skimmed milk did not. Givens [12] used cis-MUFAs instead of SFA in milk fat by altering the diet of the dairy cow, which improved indicators of coronary heart disease (CHD) and CVD in general for the consumer. Due to the ruminal biohydrogenation of dairy cows, the genetic approach is more feasible than dietary manipulation when altering the fatty acid profiles of milk [13].

MicroRNAs (miRNAs), a class of noncoding RNAs, regulate gene expression by specifically binding to 3′-untranslated regions (3′-UTRs) of their target genes [14]. MiRNAs play vital roles in widely biological processes such as growth, development, reproduction and disease [15]. Two experiments from Wang et al. [16,17] respectively demonstrated that miRNA-26a/b and miRNA-145 regulated lipogenesis by targeting the Insulin-induced gene 1 (*INSIG1*) in goat mammary epithelial cells (GMECs). Recent studies in dairy cows have shown that miRNAs participate in the regulation of lipid synthesis at a post-transcription level in dairy cows [18,19]. Lactation stage is another factor that affects the metabolite composition of milk [3]. Studies have shown that the milk fat rate in late lactation is higher than that in early lactation [20]. Therefore, we speculate that key differentially expressed microRNAs (DE miRNAs) and genes (DEGs) that regulate the synthesis of PUFAs in milk can be found by comparing the transcriptome of the mammary glands of dairy cows in two periods.

In this study, we measured the mammary gland transcriptome of dairy cows and predicted target-related miRNAs and DEGs. Then, we screened the potential target-related miRNA (miR-193a-5p) and DEG (*FADS1*) related to PUFAs synthesis. Fatty acid desaturases catalyzing SFAs to PUFAs lead to the accumulation of PUFAs in milk [21]. A study from Boschetti et al. [22] indicated that the fatty acid composition of chicken breast meat was dependent on a genotype-related variation of fatty acid desaturase 1 (*FADS1*) and fatty acid desaturase 2 (*FADS2*) and their desaturating activity. In humans, several studies suggested that plasma and tissue concentrations of n-3 and -6 fatty acids were associated with several single nucleotide polymorphisms (SNPs) in the *FADS1* gene [23]. However, the function of fatty acid desaturase 1 (*FADS1*) and miR-193a-5p, a potential regulator of *FADS1*, remains unclear in the synthesis of PUFAs in bovine milk. For the first time, we explored the regulatory relationship and molecular mechanisms of miR-193a-5p and *FADS1* in BMECs. Our study provides a reference for molecular breeding to increase the proportion of PUFAs in milk.

## 2. Materials and Methods

### 2.1. Ethics Statement

Our experiment obtained ethics approval from the Institutional Animal Care and Use Committee (IACUC) of the Yangzhou University Animal Experiments Ethics Committee (Permit Number: SYXK (Su) IACUC 2016-0019).

### 2.2. Transcriptome Sequencing

Three Holstein dairy cows (A, B, C) in their second lactation period without a history of illness were selected from the farm of Yangzhou University. The three dairy cows were feed with total mixed ration (TMR), and its components are shown in Table S1 [24]. Mammary glands of dairy cows were collected by surgical methods at early lactation (postpartum day 30, 30 d) and mid lactation (postpartum day 180, 180 d) [25,26]. Total RNA was extracted using the mirVana miRNA Isolation Kit (Ambion, Austin, TX, USA). RNA integrity was assessed on an Agilent 2100 Bioanalyzer (Agilent, Santa Clara, CA, USA), and samples with an RNA Integrity Number (RIN) ≥7 were used in transcriptome analysis.

The small RNA (sRNA) libraries were constructed according to Illumina^®^ TruSeq™ (Illumina, San Diego, CA, USA) Small RNA Sample Preparation protocol. Briefly, 3′ adaptor and 5′ adaptors were ligated to the total RNA. Then, the cDNA was obtained using reverse transcription with SuperScript II Reverse Transcriptase (Invitrogen, Carlsbad, CA, USA). Finally, the cDNA was amplified by PCR with DNA polymerase to generate small RNA libraries. The libraries were sequenced on Illumina Hiseq 2500 at a sequence read length of 1 × 36 bp to obtain the original data. The quality of raw reads was elevated using the FastQC online tool (http://www.bioinformatics. babraham.ac.uk/projects/fastqc/) [27].

A TruSeq Stranded mRNA LTSample Prep Kit (Illumina, San Diego, CA, USA) was used to construct cDNA libraries according to the manufacturer’s instructions. The libraries were sequenced on the Illumina sequencing platform (HiSeqTM 2500, Illumina, San Diego, CA, USA) to generate 125bp paired-end raw reads. Unqualified raw reads were removed to generate clean reads that were mapped to reference bovine genome UMD3.1 (ftp://ftp.ncbi.nlm.nih.gov/genomes/all/GCF_000003055.6_Bos_taurus_UMD_3.1.1) by TopHat 2.1.1 (http://ccb.jhu.edu/software/tophat/index.shtml) and Bowtie 2 2.3.5.1 (http://bowtie-bio.sourceforge.net/index.shtml) [28,29].

Datasets were obtained from the NCBI Sequence Read Archive (SRA) (https://www.ncbi.nlm.nih.gov/sra) with accessing numbers SRS6946983, SRS6946984 and SRR12149783, and the Genome Sequence Archive in the BIG Data Center, Beijing Institute of Genomics (BIG), Chinese Academy of Sciences, under accession number CRA002742, and are publicly accessible at http://bigd.big.ac.cn/gsa.

### 2.3. Identifition of Target-Related miRNA and DEG

miRNA expression was calculated as transcripts per million (TPM = reads of each miRNA alignment/reads of samples’ total alignment × 10^6^) [30]. Principal component analysis (PCA) was performed to explore transcripts’ distribution. The calculation of DE miRNAs was realized by the DEseq approach. DE miRNAs were defined as those with a fold change >2 and *p*-value < 0.05.

The transcripts’ abundance was detected using Fragment reads Per Kilobase per Million mapped reads (FPKM). The read counts of each gene were calculated by HtSeq-count 0.9.1 (https://htseq.readthedocs.io/en/master/history.html#version-0-9-1) [31]. The PCA was performed to explore transcripts’ distribution. Differential expression analysis of paired transcripts was performed using the DESeq R package (1.18.0) [32]. Genes with fold change >2 and *p*-value < 0.05 were identified as DEGs.

In addition, qRT-PCR was used for validation of the sequencing data based on the expression of DE miRNAs and DEGs. A PrimeScript™ RT Reagent Kit with a gDNA Eraser (Takara, Kyoto, Japan) was applied for the synthesis of cDNA. Expression of the DE miRNAs/DEGs was detected using the TB Green^®^ Premix Ex Taq™ II Kit (Takara, Kusatsu, Shiga, Japan)/Hairpin-it microRNA and the U6 snRNA Normalization RT-PCR Quantitation kit (GenePharma, Shanghai, China) [33]. All reactions were performed in triplicates using a Light Cycler^®^ 480 System (Roche Applied Science, Indianapolis, IN, USA). DE miRNAs/mRNAs were normalized for bovine U6/*β-actin*, and the primers used for qRT-PCR are shown in Tables S2 and S3. Relative expression was calculated using the 2^−ΔΔCt^ method for all samples [34,35].

A TargetScan program (http://www.targetscan.org) was used to identify the target gene of DE miRNAs. Only mRNAs showing a negative correlation with DE miRNAs were considered putative targets. A network map was built to investigate the interactions between DE miRNAs and their targeted DEGs. To find the target-related miRNAs and DEGs with the biological function of PUFAs synthesis, gene ontology (GO) annotation and Kyoto Encyclopedia of Genes and Genomes (KEGG) pathways analysis were respectively achieved by DAVID 6.8 (https://david.ncifcrf.gov/) [36] and KOBAS 3.0 (http://kobas.cbi.pku.edu.cn/kobas3) [37]. A *p*-value < 0.05 was the threshold for identifying significantly GO terms and KEGG pathways. In this study, miR-193a-5p and *FADS1* were considered as key DE miRNA and mRNA with the biological function of PUFAs synthesis.

### 2.4. Cell Culture and Transfection

BMECs were obtained from the Animal Genetics Laboratory of Yangzhou University. They were cultured in six-well plates with DMEM/F12 (Gibco, Waltham, MA, USA) complete media that contained 10% fetal bovine serum (FBS; Gibco), 50 U/mL penicillin/mL streptomycin, 5 mg/mL bovine insulin, 10 ng/mL EGF-1 (Gibco) and 0.25 mmol/L hydrocortisone. The plates were placed in an incubator with 5% CO_2_ at 37 °C. When BMECs grew to approximately 80% per well, the cells were transfected with miR-193a-5p mimic (100 nM final concentration) and inhibitor (200 nM final concentration) using the Lipofectamine 2000 Transfection Kit (Sigma, St. Louis, MO, USA). Transfection of BMECs was carried out in Opti-MEM medium (Gibco, Waltham, MA, USA) for 6 h. Then, Opti-MEM medium was replaced with DMEM/F12 complete media for the next 48 h of culture. The sequence of miR-193a-5p mimic, inhibitor and negative control were shown in Table S4. Each transfection held three replicates.

### 2.5. RNA Extraction and qRT-PCR

Total RNA was extracted from the transfected BMECs using TRNzol Universal Reagent Kit (Tiangen, China). RNA quality was checked by a spectrophotometer (Thermo Scientific, Waltham, MA, USA). The method mentioned in Section 2.3 was used to detect miRNA-193a-5p, mRNAs’ expression of *FADS1*, and fatty acid synthesis-related genes, including fatty acid desaturase 2 (*FADS2*), ELOVL fatty acid elongase 6 (*ELOVL6*), diacylglycerol O-acyltransferase 1 (*DGAT1*) and diacylglycerol O-acyltransferase 2 (*DGAT2*). Primers for qRT-PCR were designed based on the miR-193a-5p sequence in the miRBase database (http://www.mirbase.org) (Table S5). Primers for the detection of mRNA expression were designed based on the sequences of bovine genome in NCBI (Table S6). A protein–protein interaction (PPI) network was built to investigate the interactions of *FADS1*, *FADS2*, *ELOVL6*, *DGAT1* and *DGAT2*. The PPI network with a combined score >0.7 was built using STRING v11.0 (https://string-db.org/).

### 2.6. Western Bolt

To obtain the total protein, the transfected BMECs were lysed in RIPA buffer containing 1% PMSF (Solarbio, Beijing, China). Protein concentrations were assessed using a BCA Protein Assay Kit (Solarbio, Beijing, China). Equal amounts of protein in each well were separated on sodium dodecyl sulfate-polyacrylamide gel electrophoresis (SDS-PAGE), and then transferred to a PVDF membrane (Amersham Biosciences, Buckinghamshire, UK). Finally, the total protein was probed with the primary antibodies—monoclonal rabbit anti-FADS1 (Cell Signaling Technology, Beijing, China) and monoclonal mouse anti-β-actin (Proteintech Gronup, Shanghai, China). The secondary antibody was a polyclonal goat antirabbit HRP-conjugated IgG (Tiangen, Sichuan, China). All antibodies were used in adherence to the instructions. Signals were measured on the chemiluminescent ECL western blot system (Pierce, Appleton, WI, USA).

### 2.7. Luciferase Reporter Assay

To verify the target sites of the *FADS1* 3′ UTR and miR-193a-5p, the pmirGLO-FADS1-MUT plasmid and pmirGLO-FADS1-WT plasmid were constructed. First, a *FADS1* 3′-UTR primer containing the miR-193a-5p action site (F, 5′-CCACCTCAACTTCCAGATT-3′; R, 5′-CCTTCCCTATTCCCACA-3′) was designed and amplified using the bovine *FADS1* 3′-UTR as a template (Table S7). Next, the amplification products were inserted into a pMD18T vector (Takara, Japan), generating plasmid pMD18T-FADS1-WT. The *FADS1* target site sequence was mutated from AAGACCC to TACACGC using a Fast Site-Directed Mutagenesis Kit (Tiangen, China), generating plasmid pMD18T-FADS1-MUT. Then, the fragments of FADS1-WT/MUT were digested with Sac I and Sal I from pMD18T-FADS1-WT/MUT. Meanwhile, the pmirGLO luciferase vectors were digested with Sac I and Sal I to obtain the linearized vectors. Finally, the FADS1-WT/MUT fragments and the linearized vectors were respectively connected with DNA T4 ligase, generating pmirGLO-FADS1-MUT/WT plasmid. The pmirGLO-FADS1-MUT/WT plasmid and miR-193a-5p mimic were co-transfected into HEK 293T cells to explore whether miR-193a-5p can combine with *FADS1* 3′ UTR. After 48 h of transfection, cells were harvested and assayed for renilla fluorescence and firefly fluorescence using a Dual-Glo luciferase assay system kit (Promega Corp, Beijing, China).

### 2.8. Co-Transfection of MiR-193a-5p Inhibitor and Small RNA

The small RNA (siRNA) targeting *FADS1* and miR-193a-5p inhibitor were co-transfected into BMECs to explore whether miR-193a-5p regulates *FADS1* by targeting only *FADS1* (Table S4). The siRNA of *FADS1* and negative control siRNA were designed and synthesized by GenePharma (Suzhou, China). The sequences of siRNA were shown as follows: F, 5′-CCUUGCUGCCUGUCUACUUTT-3′; R, 5′-AAGUAGACAGGCAGCAAGGTT-3′.

### 2.9. Triglycerides Content Assay

Triglyceride was assayed using an enzymatic triglyceride assay kit (Applygen, Beijing, China). The BMECs were lysed after 48 h of transfection. The mixture was allowed to rest for 10 min, and the supernatant was transferred into a sterile 1.5 mL micro-centrifuge tube and then heated for 10 min in a 70 °C water bath. After centrifugation (5 min at 2000 rpm), the supernatant was collected for an enzymatic assay [38,39]. According to the manufacturer’s instructions, absorbance was detected at 550 nm in a microplate reader (Tecan, Switzerland).

### 2.10. Fatty Acid Profiles Assay

Intracellular fatty acids were assayed by referring to the method of Zhou et al. [40]. In detail, BMECs were transfected with miR-193a-5p mimic, inhibitor and negative control. A total of 100 mg BMECs mixed with 2 mg of methanol containing 0.25% sulfuric acid were sonicated by ultrasound and then incubated at 80 °C for 1 h to methylate the fatty acids. The solution, returned to room temperature, was mixed with 2 mL hydrochloric acid solution (0.1 M) and then 800 μL of n-hexane was added, obtaining a mixed solution. The mixed solution was centrifuged at 900× *g* for 5 min, and the supernatant was transferred to a siliconized glass tube. The supernatant was added to 0.5 g anhydrous sodium sulfate and vortexed. After centrifugation at 13,800× *g* for 5 min, the supernatant and anhydrous sodium sulfate were separated from the solution. The supernatant was applied to the detected fatty acid composition and content with gas chromatography−mass spectrometry (Thermo Fisher, Waltham, MA, USA) analysis of fatty acid composition and content. The relative content of fatty acid was evaluated using the percentage of peak area for individual fatty acids.

### 2.11. Statistical Analysis

Statistical analysis Software (SAS) 9.4 (SAS Institute, Cary, NC, USA) was used for statistical analyses. Data were presented as mean ± standard error (SE) from triplicate experiments. *p* < 0.05 indicated significant differences between the groups.

## 3. Results

### 3.1. Analysis of Differentially Expressed miRNAs and mRNA

#### 3.1.1. Identification of Differentially Expressed miRNAs and mRNAs

A total of six sRNA/cDNA libraries were established and sequenced to identify DE miRNAs/mRNAs based on the mammary glands of dairy cows during early and mid-lactation. The six sRNA/cDNA libraries produced 916.29/3913.31 million (M) raw reads (Tables S8 and S9). After removing the unqualified reads, a total of 847.55/3859.12 M clean reads were obtained from the sRNA/cDNA libraries and sequenced on Illumina Hiseq 2500. In each sRNA library, the Q20 percentage was higher than 99.88%. In each cDNA library, the Q30 percentage was greater than 96.19%, and the GC content ranges from 47.50% to 50.00%. Consequently, the sequencing results are reliable and could be used for subsequent analysis.

**Figure 1:**
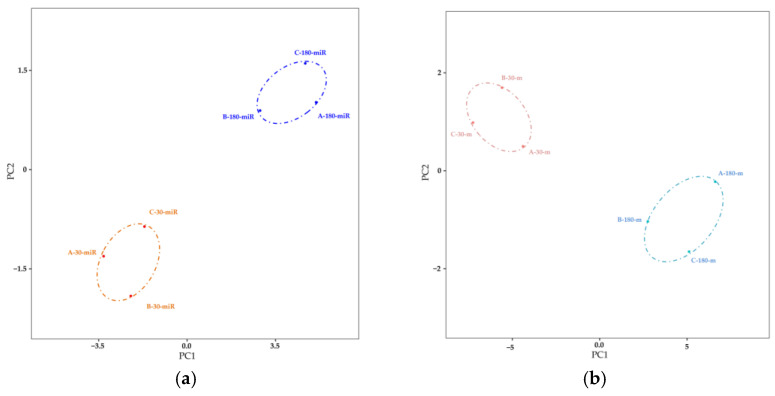
Principal component analysis of transcriptome sequencing data. (**a**) Principal component analysis of miRNA sequencing data. (**b**) Principal component analysis of mRNA sequencing data.

**Figure 2:**
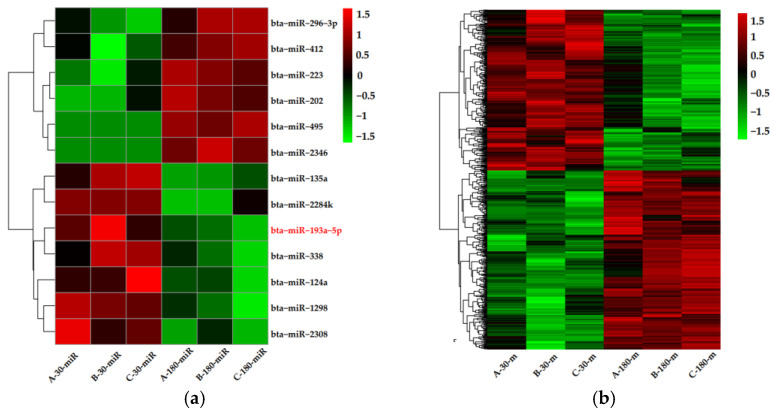
Heat maps of differentially expressed microRNAs (DE miRNAs) and genes (DEGs) in dairy cow mammary glands between early and mid-lactation. (**a**) Red modules represent upregulated DE miRNAs, and green modules represent downregulated DE miRNAs. (**b**) Red modules represent upregulated DEGs, and green modules represent downregulated DEGs.

PCA based on the sRNA/cDNA libraries was performed, respectively generating two clusters: early and mid-lactation (Figure 1). A cluster included miRNAs/mRNAs from different dairy cows who were in the same lactation stage, indicating that the main differences of the miRNA/mRNA expression profiles appeared at different stages of lactation. DE miRNAs/mRNAs were identified by comparing the miRNA/mRNA expression profiles at two time points. Subsequently, a total of 13 DE miRNAs and 632 DEGs were identified by fold change >2 and *p*-value < 0.05. The 13 DE miRNAs were 6 upregulation miRNAs and 7 downregulation miRNAs in mid-lactation, compared with early lactation (Figure 2a). The 632 DEGs include 332 upregulation genes and 300 downregulation genes in mid-lactation compared with early lactation (Figure 2b).

Additionally, the validation of sequencing data used qRT-PCR based on the expression of DE miRNAs and DEGs. The qRT-PCR results demonstrate that the expression of miR-223, miR-2346, miR-338, *FADS1*, *PHLDA2* and *SLC13A5* are upregulation in mid-lactation verse early lactation, while miR-193a-5p, miR-124a, *ACSM1*, *ACPP*, *CYP4B1*, *ACACB* and *CCDC3* are downregulation in mid-lactation verse early lactation (Tables S10 and S11). The results of qRT-PCR were in accordance with transcriptome sequencing data.

#### 3.1.2. Functional Analysis of Differentially Expressed Genes

**Figure 3:**
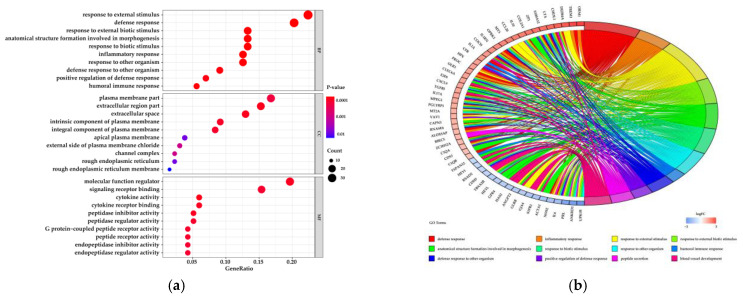

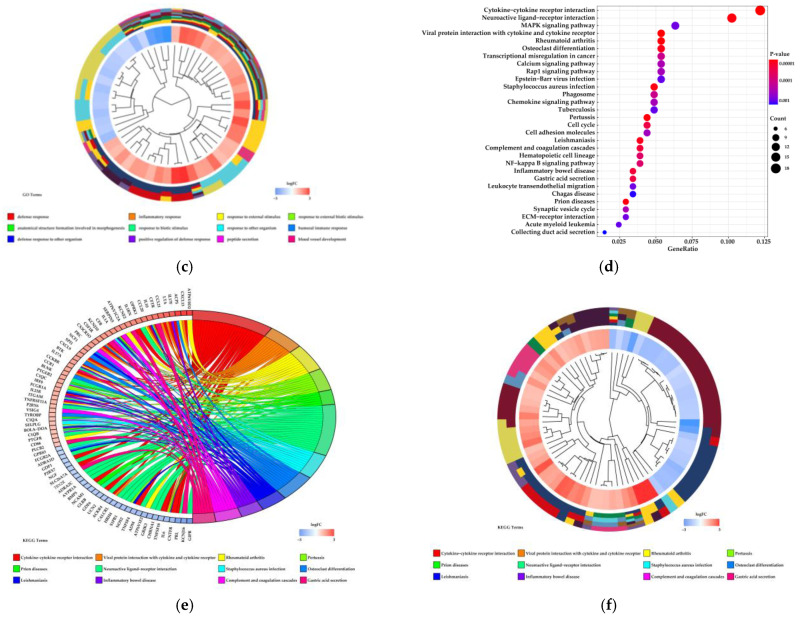
Significant gene ontology (GO) terms and Kyoto Encyclopedia of Genes and Genomes (KEGG) pathways of DEGs. (**a**) The top 10 significant GO terms are respectively listed in each catalog, including biological process (BP), cellular component (CC) and molecular function (MF). (**b**) Circos plots show specific responses and overlapping of DEGs enriched in the top 12 significant GO terms. (**c**) Circos plots indicate features of DEGs enriched in the top 12 significant GO terms. (**d**) The top 30 significant KEGG pathways of DEGs. (**e**) Circos plots show specific responses and overlapping of DEGs enriched in the top 12 significant KEGG pathways. (**f**) Circos plots indicate the features of DEGs enriched in the top 12 significant KEGG pathways.

GO annotation elucidates the biological functions of 632 DEGs better according to three classes—biological process (BP), cellular component (CC) and molecular function (MF). Figure 3a lists terms enriched significantly in each class (*p*-value < 0.05). Figure 3b,c shows changes in the expression of DEGs in the top 12 significant enrichment terms. Additionally, KEGG pathways analysis of the 632 DEGs reveals that 30 pathways show a significant difference between mid- and late lactation in the mammary glands of dairy cows (*p*-value < 0.05) (Figure 3d). The changes in DEGs of significant pathways are shown in Figure 3e,f.

### 3.2. Integration Analysis of DE miRNAs and DE mRNAs

**Figure 4:**
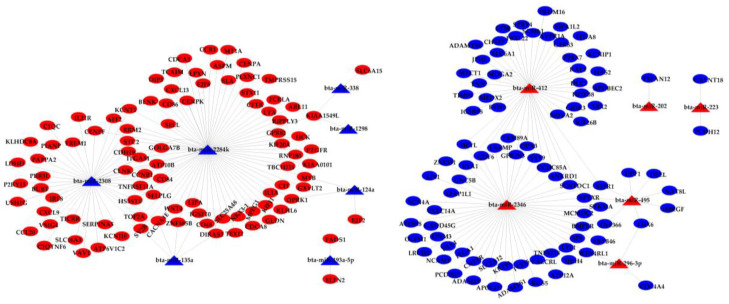
A network map of the pairs of DE miRNAs and DE mRNAs. The blue triangles represent the downregulated miRNAs, and the red triangles represent the upregulated miRNAs. The blue ellipses represent the downregulated mRNAs, and the red ellipses represent the upregulated mRNAs.

Correlation analysis of DE miRNAs and DE mRNAs found 229 negatively correlated pairs of miRNA and target mRNA. Among the miRNA–mRNA pairs, a total of 122 pairs showed that miRNA was downregulated and mRNA was upregulated in mid-lactation compared with early lactation. Bta-miR-2284k, the most-enriched miRNA, had a target of 69 DEGs. A total of 182 DEGs were regulated by 13 DE miRNAs (bta-miR-1298, bta-miR-495, bta-miR-223, bta-miR-135a, bta-miR-193a-5p, bta-miR-296-3p, bta-miR-338, bta-miR-2346, bta-miR-202, bta-miR-124a, bta-miR-124b, bta-miR-2284k, bta-miR-2308 and bta-miR-412) (Figure 4). These DE miRNAs–mRNAs pairs may play an important role in milk fatty acid synthesis in dairy cows.

### 3.3. MiR-193a-5p Specific Targeting of FADS1

**Figure 5:**
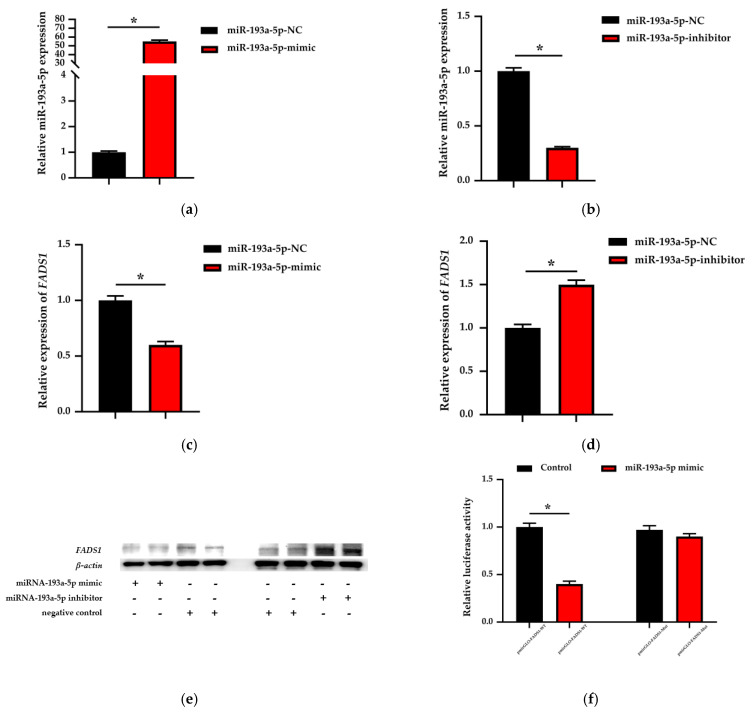
Relative expression of miRNA-193a-5p in BMECs after transfecting the (**a**) mimic and (**b**) inhibitor for 48 h, and the relative mRNA expression of *FADS1* after transfecting the (**c**) mimic and (**d**) inhibitor of miRNA-193a-5p for 48 h (*n* = 9). NC represents the negative control. (**e**) Relative expression of the *FADS1* protein in BMECs after transfecting the mimic and inhibitor of miRNA-193a-5p for 48 h. (**f**) Relative luciferase activity in BMECs that transfected and co-transfected with miR-193a-5p mimic and pmirGLO-FADS1-WT/pmirGLO-FADS1-MUT for 48 h (*n* = 9). Data are presented as mean ± standard error (SE). * represents *p* < 0.05, indicating significant differences between the groups.

Given that the function of the fatty acid metabolism of *FADS1* remains to be fully clarified in BMECs, we selected the miR-193a-5p–*FADS1* candidate pair and attempted to verify their biological function and regulatory relationships. The BMECs were transfected with miR-193a-5p mimic, inhibitor and miRNA negative control. After 48 h, the expression of miR-193a-5p was 55 times greater in the mimic group compared with the negative control (Figure 5a). In contrast, compared with the negative control, the expression of miR-193a-5p was more than 70% lower in BMECs whose miRNA-193a-5p was silenced (Figure 5b). The results were successful for the transfection of miR-193a-5p mimic and inhibitor.

Figure 5c–e shows the expression of *FADS1* in BMECs transfected with mimic and an inhibitor of miR-193a-5p. Both mRNA and protein results indicate that *FADS1* expression is upregulated in BMECs transfected by the inhibitor of miR-193a-5p, while the overexpression of miR-193a-5p leads to the downregulation of *FADS1* expression in BMECs.

Dual luciferase reporter gene assay evaluated whether miR-193a-5p targets *FADS1* in BMECs. We predicted the 3′ UTR region of *FADS1* in dairy cows using TargetScan and constructed wild-type (pmirGLO-FADS1-WT) and mutant (pmirGLO-FADS1-MUT) vector plasmids. The two types of vectors were co-transfected with miR-193a-5p mimic into BMECs. In the group of co-transfections with pmirGLO-FADS1-WT and miR-193a-5p mimic, luciferase activity significantly decreased to 40% of the negative control group (Figure 5f). In the group of co-transfections with pmirGLO-FADS1-MUT and miR-193a-5p mimic, luciferase activity had no statistical significance compared with the negative control group (Figure 5f). These results confirm that miR-193a-5p regulates *FADS1* expression by binding to the predicted target site in the 3′ UTR region of *FADS1* in dairy cows.

### 3.4. Triglycerides Content Analysis

**Figure 6:**
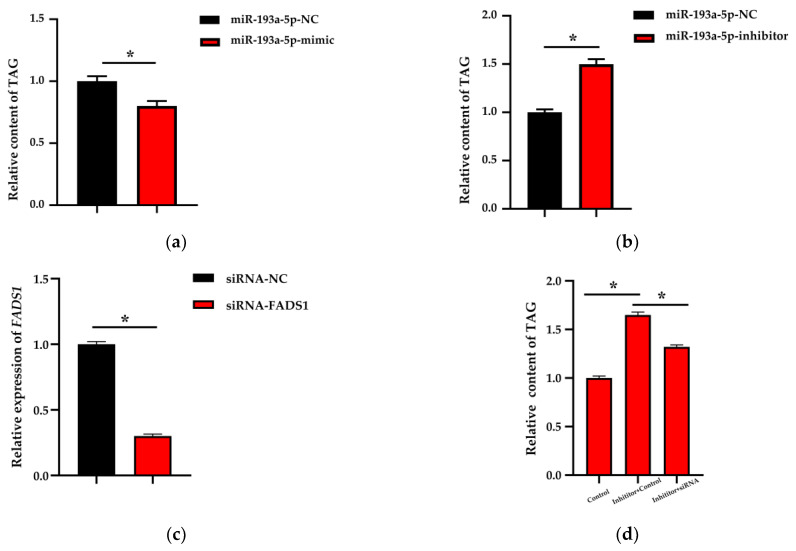
The relative content of triglycerides (TAG) in BMECs after transfecting with the (**a**) mimic and (**b**) inhibitor (*n* = 9). (**c**) The relative mRNA expression of *FADS1* after transfecting siRNA for 48 h. (**d**) The relative content of TAG in BMECs after co-transfecting with siRNA and the inhibitor of miRNA-193a-5p for 48 h. NC represents the negative control. Data are presented as mean ± standard error (SE). * represents *p* < 0.05, indicating significant differences between the groups.

After confirming the regulation relationships between miR-193a-5p and *FADS1*, we sought to explore the special biological function of the miR-193a-5p–*FADS1* candidate pair in BMECs. As triglycerides (TAG) is the main composition of milk fat and the main carrier of milk fatty acids, we examined the TAG levels in BMECs transfected with miR-193a-5p mimic/inhibitor. Compared with the controls, the miR-193a-5p mimics reduced the TAG level to 20% (*p* < 0.05) (Figure 6a). In contrast, TAG levels increased to 150% when miR-193a-5p was inhibited in BMECs (Figure 6b).

### 3.5. Partial Rescue of the Triglycerides Reduction

The experiments have indicated that miR-193a-5p regulates *FADS1* expression by binding to the predicted target site in the 3′ UTR region of *FADS1*, and miR-193a-5p inhibits the synthesis of cellular TAG. A rescue experiment was performed to verify whether miR-193a-5p induces the increase of TAG levels by targeting *FADS1*. Rescues of siRNA-FADS1 increase the TAG levels in BMECs. siRNA-FADS1 partially alleviated the increase in TAG content in BMECs treated with miR-193a-5p inhibitor (Figure 6d). The results demonstrate that miR-193a-5p induces an increase in TAG levels by targeting *FADS1*.

### 3.6. Fatty Acid Profiles Analysis

**Figure 7:**
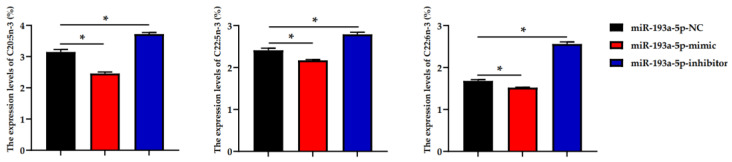
Effect of miR-193a-5p mimic and inhibitor on the composition of n-3 fatty acids in BMECs (*n* = 6). * represents *p* < 0.05, indicating significant differences between the groups.

To further understand the role of miRNA-193a-5p in the fatty acid metabolism of BMECs, we detected changes in the fatty acid content of BMECs transfected with miR-193a-5p mimic/inhibitor. BMECs transfected with miR-193a-5p inhibitor show an increase in concentration of PUFAs such as eicosapentaenoic acid (EPA, C20:5n-3), docosapentaenoic acid (DPA, C22:5n-3) and docosahexaenoic acid (DHA, C22:6n-3) compared with the control group (Table 1). BMECs transfected with miR-193a-5p mimic show a decrease in concentration of PUFAs such as EPA, DPA and DHA compared with the control group (Figure 7).

### 3.7. Expression Analysis of Fatty Acid Metabolism-Related Genes

**Figure 8:**
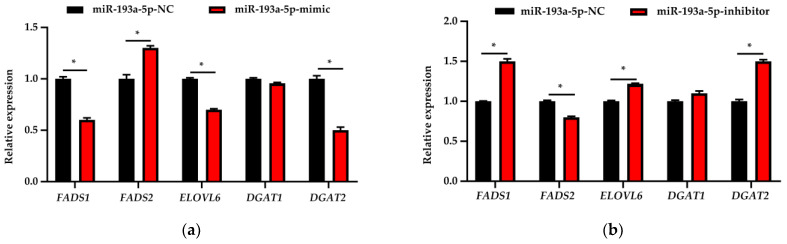

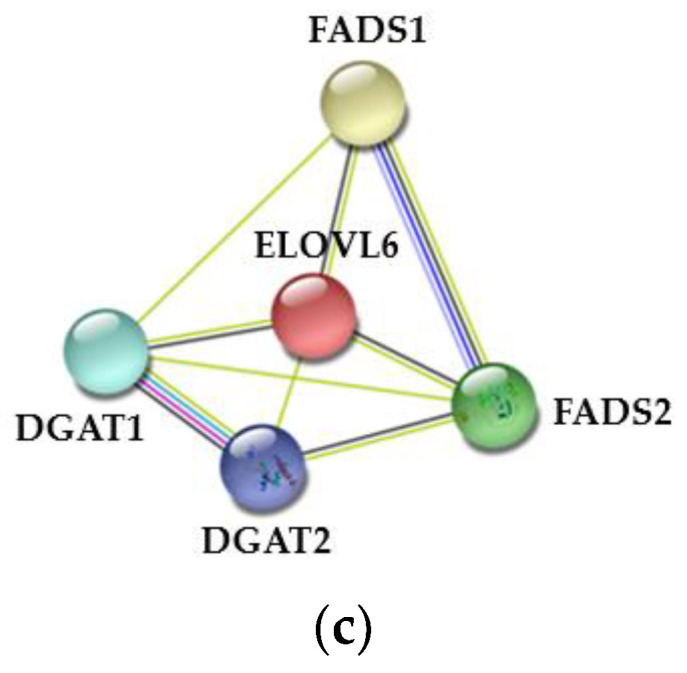
Relative expression of genes related to fatty acid metabolism after transfecting with the (**a**) mimic and (**b**) inhibitor (*n* = 9). NC represents the negative control. Data were presented as mean ± standard error (SE). * represents *p* < 0.05, indicating significant differences between the groups. (**c**) A protein–protein interaction (PPI) network of *FADS1* and genes related to fatty acid metabolism, including *FADS2*, *ELOVL6*, *DGAT1*, *DGAT2*.

MiR-193a-5p overexpression reduces the expression of *FADS1* and other fatty acid metabolism-related genes in BMECs, including *ELOVL6* and *DGAT2*, and upregulates the expression of *FADS2*, an isozyme of *FADS1* (*p* < 0.05) (Figure 8a). Inhibition of miR-193a-5p increases the expression of *FADS1*, *ELOVL6* and *DGAT2* and suppresses *FADS2* expression in BMECs (*p* < 0.05) (Figure 8b). *DGAT1* expression is not affected by the expression of miR-193a-5p and *FADS1* (*p* > 0.05).

## 4. Discussion

Research showed that *FADS1* encodes rate-limiting enzymes in the endogenous formation of PUFAs [41,42]. EPA and DHA are endogenous produced in a pathway where essential n-3 fatty acids and α—linolenic acid are desaturated and elongated. In this pathway, *FADS1* plays a critical role [43,44]. Approximately 17.9 million people die from CVD every year [45,46]. Increasing evidence shows that nutrition plays a pivotal role in the development of chronic diseases, especially CVD [47]. Diet and lifestyle are the key risk factors for CVD prevention and have been the focus of intense research [46]. Dairy products, as an integral part of human nutrition, contain n-3 fatty acids, usually at concentrations below biological requirements. Thus, in recent years, some research has been devoted to increasing the content of PUFAs in bovine milk based on genome, transcription and post-transcriptional levels [48,49,50]. In this study, we confirmed that miR-193a-5p directly binds to the *FADS1* 3′-UTR in dairy cows. qRT-PCR and western bolt assays indicated that overexpression of miR-193a-5p downregulated expressions of *FADS1* regardless of mRNA or protein levels, and the inhibition of miR-193a-5p had the opposite effect. In addition, the content of TAG, EPA, DPA and DHA has a significant positive correlation with the expression of *FADS1* and a significant negative correlation with the expression of miR-193a-5p in BMECs. These results confirm that miR-193a-5p regulates the synthesis of PUFAs by targeting *FADS1* in BMECs. Increasing PUFAs intake is beneficial for global health and can prevent human beings from metabolic, cardiovascular, cognitive and developmental health conditions [51,52,53]. From a global health perspective, the downregulation of miRNA-193a-5p and the upregulation of *FADS1* are beneficial.

Polymorphisms in *FADS1*- and *FADS2*-altered fatty acid profiles or the activity of *FADS1*- and *FADS2*-. He et al. [54] identified strong associations between SNPs in the fatty acid desaturase (*FADS*) region and multiple PUFAs, suggesting that genetic variants in the *FADS* region were major genetic modifiers that could regulate fatty acid metabolism through epigenetic gene regulation. Koletzko et al. [55] found that genotype distribution differs markedly among ethnicities, apparently reflecting an evolutionary advantage of genotypes in activating LC-PUFAs synthesis when the introduction of agriculture provided diets rich in LA but with little arachidonic acid (AA) and EPA. Vegetarians with a TT genotype of the rs174547 in *FADS1* gene had higher odds of developing metabolic syndrome (MetS), larger waist circumference (WC), higher blood pressure (BP), and lower level of HDL-c, which suggested that the dietary intake of LA might interact with rs174547 in the *FADS1* gene and affect HDL-c levels [56]. Joshi et al. [57] demonstrated that an increase the *FADS1* expression of vegetarian pregnant women during pregnancy might maintain AA, EPA and DHA levels to ensure the overall LC-PUFA levels of the neonate. Populations had an increase in EPA in plasma when intake of flaxseed oil rich in α-linolenic acids (ALA) increased beyond that of major allele homozygotes consuming a typical “western” diet [58]. Individuals homozygous for the minor allele of *FADS1*/*FADS2* had a lower plasma AA and AA/LA ratio when compared with the major allele carriers after each diet, while ELOVL fatty acid elongase 2 (*ELOVL2*) had no effect on PUFAs [58]. Their findings provided strong supporting evidence for the role of FADS1 in regulating PUFAs metabolism. In our study, no SNPs were found in *FADS1* 3′ UTR. The inhibitor of miR-193a-5p significantly increased *FADS1* expression, either mRNA or protein, which upregulated the n-3 fatty acid levels. Our experimental results further clarify that miR-193a-5p plays an important role in the regulation of milk fatty acid profiles at a post-transcriptional level.

**Figure 9:**
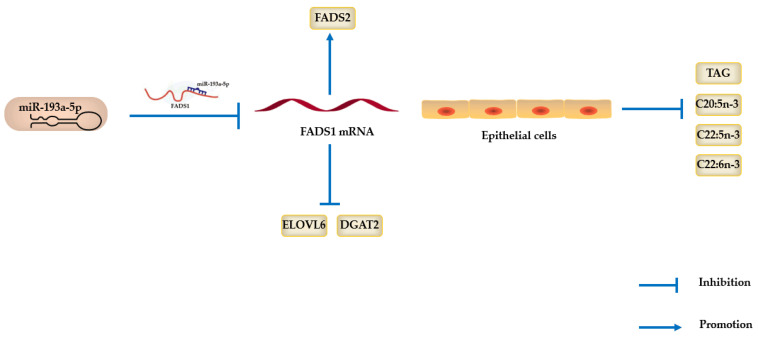
MiRNA-193a-5p regulated the synthesis of eicosapentaenoic acid (EPA), docosapentaenoic acid (DPA) and docosahexaenoic acid (DHA) by targeting *FADS1* in BMECs. After transfecting the miRNA-193a-5p mimics into BMECs, *FADS1* expression was downregulated, which led to the upregulation of *FADS2* and the downregulation of *ELOVL6* and *DGAT2*.

According to the regulatory relationship network (Figure 9), miRNA-193a-5p regulates EPA, DPA and DHA synthesis by targeting *FADS1* in BMECs. In PPI and regulatory relationship networks, *FADS1* respectively interacts with *FADS2*, *ELOVL6* and *DGAT1*, and the expression of *FADS1* could influence the expression of *DGAT2* through *FADS2*, *ELOVL6* and *DGAT1*. As is well known, *ELOVL6* is a key enzyme in intracellular lipid metabolism [59]. Du et al. [60] found that *ELOVL6* was identified as a target gene of miR-125a-5p during porcine intramuscular adipogenesis, and that the overexpression of miR-125a-5p decreased the content ratios of monounsaturated fatty acids (MUFAs) and total SFAs, while having no significant impact on PUFAs/SFAs and n-6/n-3 ratios. Takamura et al. [61] proved that the knockout of *ELOVL6* in mice could inhibit lipid accumulation and increase cholesterol consumption. Shi et al. [48,62] demonstrated that *ELOVL5* and *ELOVL7* play roles in the synthesis of long-chain unsaturated fatty acids (LC-UFAs) in goat mammary epithelial cells. In the current study, *ELOVL6* expression shows a positive correlation with *FADS1* expression and a negative correlation with miR-193a-5p expression. Also, *DGAT2* expression shows a positively correlation with *FADS1* expression and a negative correlation with miR-193a-5p expression. *DGAT2*, belonging to the *DGAT* gene superfamily, catalyzes the final, committed step in acyl-CoA-dependent TAG biosynthesis [63]. Stone et al. [64] confirmed that *DGAT2* plays a fundamental role in mammalian TAG synthesis, and *DGAT1* plays an important part in regulating energy metabolism. In addition, multiple studies concluded that *DGAT1* and *DGAT2* share no homology [64,65,66]. In our study, the expression of miR-193a-5p and *FADS1* have statistical significance with *DGAT1*.

The downregulation of miRNA-193a-5p and upregulation of *FADS1* significantly increased the content of dairy fat in BMECs. Some studies are skeptical about the safety of high-fat milk intake [67]. An early study reported that a strong correlation existed between dairy fat consumption and CHD [68]. However, in recent years, a growing number of studies have found no significant correlation between milk fat content and CVDs [5]. The effect of dairy fat on health is demonstrated by the fatty acid profile [16,69,70]. Thus, improving the fatty acid profile of dairy fat continues to be a worthy topic for future studies.

## 5. Conclusions

For the first time, our data identify that miRNA-193a-5p regulates PUFAs metabolism in BMECs by targeting *FADS1*, suggesting that miRNA-193a-5p and *FADS1* are underlying factors for improving PUFAs content in bovine milk (Figure 9).

## Figures and Tables

**Table 1:** Effects of miR-193a-5p mimic and inhibitor on intracellular fatty acid composition in BMECs.

Fatty Acid (%)	Treatment		
	NC	miR-193a-5p-Mimic	miR-193a-5p-Inhibitor
C12:0	0.16 ± 0.02	0.15 ± 0.02	0.15 ± 0.02
C14:0	1.51 ± 0.03 c	1.59 ± 0.05 b	1.66 ± 0.04 a
C16:0	21.31 ± 0.26 b	23.29 ± 0.32 a	21.33 ± 0.35 b
C18:0	27.16 ± 0.51 a	25.20 ± 0.38 b	27.38 ± 0.52 a
C16:1	1.58 ± 0.03	1.56 ± 0.04	1.53 ± 0.02
Cis-9-C18:1	21.05 ± 0.46	21.01 ± 0.34	21.09 ± 0.37
C18:1	14.64 ± 0.24 b	15.25 ± 0.13 a	13.11 ± 0.17 c
C18:2	2.26 ± 0.05	2.23 ± 0.04	2.21 ± 0.03
C20:4n-3	3.09 ± 0.07 b	3.57 ± 0.11 a	2.47 ± 0.06 c
C20:5n-3	3.15 ± 0.08 b	2.46 ± 0.05 c	3.72 ± 0.05 a
C22:5n-3	2.41 ± 0.05 b	2.17 ± 0.02 c	2.79 ± 0.05 a
C22:6n-3	1.68 ± 0.03 b	1.52 ± 0.01 c	2.56 ± 0.05 a

Note: a, b, c = *p* < 0.05 in the row (*n* = 6).

## Data Availability

The data presented in this study are available from supplementary materials.

## Supplementary Materials

The following are available online at https://www.mdpi.com/2218-273X/11/2/157/s1. Table S1. Composition and nutrient levels of total mixed ration (TMR). Table S2. The primers used for qRT-PCR to validate the Small RNA sequencing. Table S3. The primers used for qRT-PCR to validate the RNA sequencing. Table S4. The sequences to alter miR-193a-5p and *FADS1* expression in BMECs. Table S5. The primers used for qRT-PCR to detect miR-193a-5p expression in BMECs. Table S6. The primers used for qRT-PCR to detect the expression of *FADS1* and other genes related to fatty acid metabolism in BMECs. Table S7. A primer containing the miR-193a-5p action site. Table S8. Statistics of miRNA sequencing reads. Table S9. Statistics of mRNA sequencing reads. Table S10. Verification results of Small RNA sequencing by qRT-PCR. Table S11. Verification results of RNA sequencing by qRT-PCR.
