# Supplementary material for: MicroRNA-193a-5p Regulates the Synthesis of Polyunsaturated Fatty Acids by Targeting Fatty Acid Desaturase 1 (FADS1) in Bovine Mammary Epithelial Cells
Source: Biomolecules. 2021 Jan 25;11(2):157. doi: 10.3390/biom11020157 (PMC7911131; doi:10.3390/biom11020157)
Supplement: Supplementary file 1 [file biomolecules-11-00157-s001.pdf]

Table S1. Composition and nutrient levels of total mixed ration (TMR).

Ingredients	Diet (%)	Nutrient levels	Diet
Corn	62.00	NE(MJ/kg)	7.94
Soybean meal	12.00	CP	12.97
Cottonseed meal	8.00	Ca	0.60
$CaHPO_4$	1.50	P	0.64
NaCl	0.30		
Premix	0.70		
Barley	22.00		
Fat	1.00		
Soda	0.50		

NE: net energy.

Table S2. The primers used for qRT-PCR to validate the Small RNA sequencing.

miRNA	Forward Primers (5′-3′)
bta-U6-F	CGCTTCGGCAGCACATATAC
bta-U6-R	TTCACGAATTTGCGTGCGTGTCATC
bta-miR-193a-5p-F	ACATTGGGTCTTTGCGGG
bta-miR-193a-5p-R	CACTTCCTCAGCACTTGTTCCTAT
bta-miR-124a-F	CGCGTAAGGCACGCGGTGAAT
bta-miR-124a-R	ATCCAGTGCAGGGTCCGAGG
bta-miR-223-F	TGGACGGAGAACTGATAAGGGTAAA
bta-miR-223-R	GAATGGATTTTTGGAGCAGGAA
bta-miR-2346-F	GCGGCGGACTGATGTGAAGG
bta-miR-2346-R	ATCCAGTGCAGGGTCCGAGG
bta-miR-338-F	GCCGCTCCAGCATCAGTG
bta-miR-338-R	GCAGGGTCCGAGGTATTC

Table S3. The primers used for qRT-PCR to validate the RNA sequencing.

Gene	Forward Primers (5′-3′)	Reverse Primers (5′-3′)	Length (bp)	GenBank ID
β-actin	CATCCTGACCCTCAAGTA	CTCGTTGTAGAAGGTGTG	91	NM_173979.3
ACSM1	ATAAGCTGTGGCACTCTCCG	TTTCCTTCCGTGTTGGGAGG	125	NM_174682
FADS1	CTCAGCCTTCGCTGACATTG	CTGCTTTGGTGCCATAACCC	118	XM 005226961
PHLDA2	TGTGCGGGCAAGCTACATAA	TGGAATGGTGGTGGAGTACA	101	NM 001076521
ACPP	TGTTTCACTGCTGTTGTCAT	GCAGGAAACTTGAAGATTCG	105	NM_001098866
CYP4B1	GTTCGAAGGCCATGACACCA7	TCCCACTTCAAAGTGTCCCG	135	NM_001076202
SLC13A5	ATGATGGTGCCCATCGTGGAG	AAAGTTGTTTGGTTCCCTGGCA	134	NM_001191446
ACACB	CTGAACATCGTGGACGTGGA	CTCGATGTGAGAGCCGTTCA	111	NM_001205333
CCDC3	CTTCCCAGGGTGTGAAAAGG	AGCCGCTACCAAATGCAGAG	95	NM_001172375

Table S4. The sequences to alter miR-193a-5p and *FADS1* expression in BMECs.

**Name**	**Sense (5′-3′)**	**Antisense (5′-3′)**
miR-193a-5p mimics	UGGGUCUUUGCGGGCGAGAUGA	AUCUCGCCCGCAAAGACCCAUU
miR-193a-5p inhibitor	UCAUCUCGCCCGCAAAGACCCA	
Negative control	CAGUACUUUUGUGUAGUACAA	
siRNA-FADS1	CCUUGCUGCCUGUCUACUUTT	AAGUAGACAGGCAGCAAGGTT
siRNA-NC	UUCUCCGAACGUGUCACGUTT	ACGUGACACGUUCGGAGAATT

Table S5. The primers used for qRT-PCR to detect miR-193a-5p expression in BMECs.

miRNA	Forward Primers (5′-3′)
bta-U6-F	CGCTTCGGCAGCACATATAC
bta-U6-R	TTCACGAATTTGCGTGCGTGTCATC
bta-miR-193a-5p-F	ACATTGGGTCTTTGCGGG
bta-miR-193a-5p-R	CACTTCCTCAGCACTTGTTCCTAT

Table S6. The primers used for qRT-PCR to detect the expression of *FADS1* and other genes related to fatty acid metabolism in BMECs.

Gene	Forward Primers (5′-3′)	Reverse Primers (5′-3′)	Length (bp)	GenBank ID
β-actin	CATCCTGACCCTCAAGTA	CTCGTTGTAGAAGGTGTG	91	NM_173979.3
FADS1	CTCAGCCTTCGCTGACATTG	CTGCTTTGGTGCCATAACCC	118	XM 005226961
FADS2	TGGTTTCGAGGCATTCACG	AAGAGGGTGGTATGAAGCCTG	131	NM 001083444
ELOVL6	AAGGTTACGGGTTGTAGCCG	ACTAGACCGAGGCTGTGCTA	125	NM 001102155
DGAT1	CCACTGGGACCTGAGGTGTC	GCATCACCACACACCAATTCA	101	XM 005688895
DGAT2	CATGTACACATTCTGCACCGATT	TGACCTCCTGCCACCTTTCT	100	NM_205793

Table S7. A primer containing the miR-193a-5p action site.

Gene	Forward Primers (5′-3′)	Reverse Primers (5′-3′)	Length (bp)
FADS1-miR-193a-5p	CCACCTCAACTTCCAGATT	CCTTCCCTATTCCCACA	520

Table S8. Statistics of miRNA sequencing reads.

Samples	Raw reads	Reads trimmed length	Q20	Clean reads
A-30-miR	12,477,545	11,668,112	99.91 %	11,422,970
B-30-miR	12,628,938	11,817,801	99.90 %	11,570,940
C-30-miR	19,597,313	18,634,520	99.89 %	18,589,700
A-180-miR	13,510,036	12,199,892	99.87 %	12,154,411
B-180-miR	15,388,298	14,131,975	99.89 %	14,080,925
C-180-miR	18,027,045	16,976,491	99.88 %	16,935,769

Table S9. Statistics of mRNA sequencing reads.

Samples	Raw reads	Raw bases	Clean reads	Clean bases	Valid bases	Q30	CG
A-30-m	60,909,716	7,613,714,500	60,088,626	7,509,331,405	98.62%	97.07%	49.00%
B-30-m	56,006,056	7,000,757,000	55,369,090	6,919,663,405	98.84%	97.27%	47.50%
C-30-m	56,573,916	7,071,739,500	55,890,988	6,984,780,894	98.77%	97.18%	48.00%
A-180-m	73,996,266	9,249,533,250	72,947,722	9,117,045,311	98.56%	96.54%	48.00%
B-180-m	67,909,316	8,488,664,500	66,910,040	8,362,357,880	98.51%	96.33%	49.00%
C-180-m	75,935,580	9,491,947,500	74,705,698	9,336,502,305	98.36%	96.19%	50.00%

Table S10. Verification results of Small RNA sequencing by qRT-PCR.

miRNA	Small RNA-seq	qRT-PCR
	**Log_2(fold change)**	**Log_2(fold change)**
bta-miR-193a-5p	-1.71	-1.47
bta-miR-124a	-1.87	-1.98
bta-miR-223	+2.36	+2.65
bta-miR-2346	+1.93	+1.71
bta-miR-338	+1.01	+1.32

Table S11. Verification results of RNA sequencing by qRT-PCR.

Gene	RNA-seq	qRT-PCR
	**Log_2(fold change)**	**Log_2(fold change)**
ACSM1	-3.02	-3.35
FADS1	+1.71	+1.37
PHLDA2	+1.45	+1.69
ACPP	-2.93	-2.16
CYP4B1	-4.12	-5.23
SLC13A5	+1.57	+1.79
ACACB	-1.16	-1.41
CCDC3	-1.32	-1.56
